# Relationship between Telomere Length, *TERT* Genetic Variability and *TERT*, *TP53*, *SP1*, *MYC* Gene Co-Expression in the Clinicopathological Profile of Breast Cancer

**DOI:** 10.3390/ijms23095164

**Published:** 2022-05-05

**Authors:** Marta Dratwa, Barbara Wysoczanska, Wioletta Brankiewicz, Martyna Stachowicz-Suhs, Joanna Wietrzyk, Rafał Matkowski, Marcin Ekiert, Jolanta Szelachowska, Adam Maciejczyk, Mariusz Szajewski, Maciej Baginski, Katarzyna Bogunia-Kubik

**Affiliations:** 1 Laboratory of Clinical Immunogenetics and Pharmacogenetics, Hirszfeld Institute of Immunology and Experimental Therapy, Polish Academy of Sciences, 53-114 Wroclaw, Poland; barbara.wysoczanska@hirszfeld.pl; 2 Department of Pharmaceutical Technology and Biochemistry Faculty of Chemistry, Gdansk University of Technology, 80-233 Gdansk, Poland; s174618@student.pg.edu.pl (W.B.); maciej.baginski@pg.edu.pl (M.B.); 3 Department of Experimental Oncology, Hirszfeld Institute of Immunology and Experimental Therapy, Polish Academy of Sciences, 53-114 Wroclaw, Poland; martyna.stachowicz@hirszfeld.pl (M.S.-S.); joanna.wietrzyk@hirszfeld.pl (J.W.); 4 Breast Unit, Lower Silesian Oncology, Pulmonology and Hematology Center, 53-413 Wroclaw, Poland; rafal.matkowski@umw.edu.pl (R.M.); marcin.ekiert@umw.edu.pl (M.E.); jolanta.szelachowska@umw.edu.pl (J.S.); adam.maciejczyk@umw.edu.pl (A.M.); 5 Department of Oncology, Wroclaw Medical University, 53-413 Wroclaw, Poland; 6 Department of Oncological Surgery, Gdynia Oncology Centre, 81-519 Gdynia, Poland; mszajewski@szpital-morski.pl; 7 Division of Propaedeutics of Oncology, Medical University of Gdansk, 80-210 Gdansk, Poland

**Keywords:** breast cancer telomerase reverse transcriptase (*TERT*), telomere length, expression of transcription factors genes, single nucleotide polymorphism (SNP)

## Abstract

The molecular mechanisms of telomerase reverse transcriptase (*TERT)* upregulation in breast cancer (BC) are complex. We compared genetic variability within *TERT* and telomere length with the clinical data of patients with BC. Additionally, we assessed the expression of the *TERT*, *MYC*, *TP53* and *SP1* genes in BC patients and in BC organoids (3D cell cultures obtained from breast cancer tissues). We observed the same correlation in the blood of BC patients and in BC organoids between the expression of *TERT* and *TP53*. Only in BC patients was a correlation found between the expression of the *TERT* and *MYC* genes and between *TP53* and *MYC*. We found associations between *TERT* genotypes (rs2735940 and rs10069690) and *TP53* expression and telomere length. BC patients with the *TT* genotype rs2735940 have a shorter telomere length, but patients with *A* allele rs10069690 have a longer telomere length. BC patients with a short allele VNTR-MNS16A showed higher expression of the *SP1* and had a longer telomere. Our results bring new insight into the regulation of *TERT*, *MYC*, *TP53* and *SP1* gene expression related to *TERT* genetic variability and telomere length. Our study also showed for the first time a similar relationship in the expression of the above genes in BC patients and in BC organoids. These findings suggest that *TERT* genetic variability, expression and telomere length might be useful biomarkers for BC, but their prognostic value may vary depending on the clinical parameters of BC patients and tumor aggressiveness.

## 1. Introduction

Breast cancer (BC) is the most common malignant tumor neoplasm in women worldwide [1]. About ten percent of BC cases are associated with a genetic predisposition or family history, with variations by country and ethnicity [2]. BC is a heterogeneous and polygenic disease, and treatment strategies vary depending on the molecular subtype as well as the most common differentially expressed genes that exist in different disease subtypes [3].

The relationship between telomerase reverse transcriptase (*TERT*) and the risk of BC has been investigated in several publications in the contexts of gene polymorphism, telomere length and the mechanism of gene expression regulation [4]. Various mechanisms, including genetic mutations and epigenetic changes, have been proposed to explain the pleiotropic association of the 5p15.33 region in which the *TERT* gene resides with telomerase activity and cancer predisposition [5,6].

The *TERT* gene encodes the catalytic subunit of telomerase, which is a key enzyme for the maintenance of telomere length; therefore, genetic variations in this region likely influence BC risk through multiple distinct biological pathways, with telomere length being only one of the implied mechanisms [7,8]. The upregulation of the *TERT* gene in BC leads to the activation of telomerase, which contributes to the growth advantage and survival of tumor cells. The molecular mechanisms of *TERT* upregulation are complex, tumor subtype specific and may be clinically relevant [9,10]. The transcriptional regulation of the *TERT* gene is a complex process, and several mechanisms that may play a role have been described, including mutations in the *TERT* promoter that can alter the binding sites of transcription factors, e.g., MYC, SP1 and ETS family proteins [11,12].

In BC, mutation of the *TERT* promoter is rare; therefore, other genetic changes have been described such as gene amplification and the presence of gene copy number gains or single nucleotide polymorphisms (SNPs), which may play a regulatory function in *TERT* expression and be associated with different telomere lengths [13,14,15].

The present study investigated the relationship between *TERT* gene polymorphisms, both SNPs and a variable number of tandem repeats (VNTR), in the context of mRNA *TERT* gene expression and telomere length and clinical parameters in female patients with BC. Additionally, we assessed the expression of the *TERT*, *MYC*, *TP53* and *SP1* genes in patients with BC and in BC organoids.

In our study, the same correlation was found between the relative expression of *TERT* and *TP53* in the whole blood of BC patients and in BC organoids. Moreover, we observed that the two *TERT* polymorphisms (rs2735940 and rs10069690) correlated with *TP53* expression and telomere length. Additionally, BC patients with a short allele (S) within VNTR-MNS16A showed higher expression of the *SP1* and had longer telomeres. Our results provide more information on the regulation of *TERT* in terms of mRNA expression as well as the genetic variability of *TERT* and telomere length in patients with BC. We have also shown that the *TERT* related genes *MYC*, *TP53* and *SP1* play an important role in BC carcinogenesis.

## 2. Results

### 2.1. Disparities of Single Nucleotide and VNTR-MNS16A TERT Gene Polymorphisms in BC

**Figure 1:**
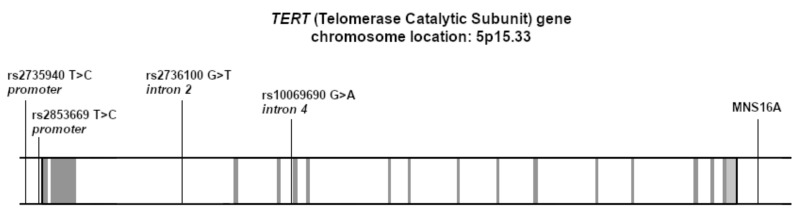
Genomic structure of the human telomerase TERT gene and the location of the studied SNPs and VNTR polymorphism. The exons are shown in grey, while the intronic regions are in white.

BC patients and healthy individuals were genotyped for TERT single nucleotide polymorphism (SNP; rs10069690, rs2735940, rs2736100 and rs2853669) and variable number tandem repeats MNS16A (VNTR-MNS16A). Their location in the TERT gene is shown in Figure 1. The genotype frequencies for all the SNPs were consistent with the Hardy–Weinberg equilibrium in both study groups. Table 1 shows the distribution of the TERT genotypes in our study group (BC women) and the control group (healthy women) and the frequency of these polymorphisms in the European population (using data from the Ensembl database, accessed on 2 February 2022). There was no difference in the distribution of alleles and genotypes between BC patients and healthy controls in any of the SNPs tested.

Four different VNTR-MNS16A alleles were detected in our BC patients and in the healthy controls (VNTR-333, VNTR-302, VNTR-274 and VNTR-234; Table 2). Patients with BC carried eight different genotypes (long (LL): 302/302, 302/333; short/long (SL): 243/302, 243/333, 274/302; short (SS): 243/243, 274/274, 243/274), but seven genotypes were noted in the control group (no 274/274 genotype as compared to BC patients). The tandem repeats rates were consistent with the Hardy–Weinberg equilibrium in the patients group, but an imbalance was observed in healthy subjects (Table 2). BC patients and healthy individuals showed no significant differences in the VNTR-MNS16A genotypes and allele frequencies.

### 2.2. Relationships between the Expression of TERT, SP1, MYC and TP53 Genes in BC Patients and BC Organoids

**Figure 2:**
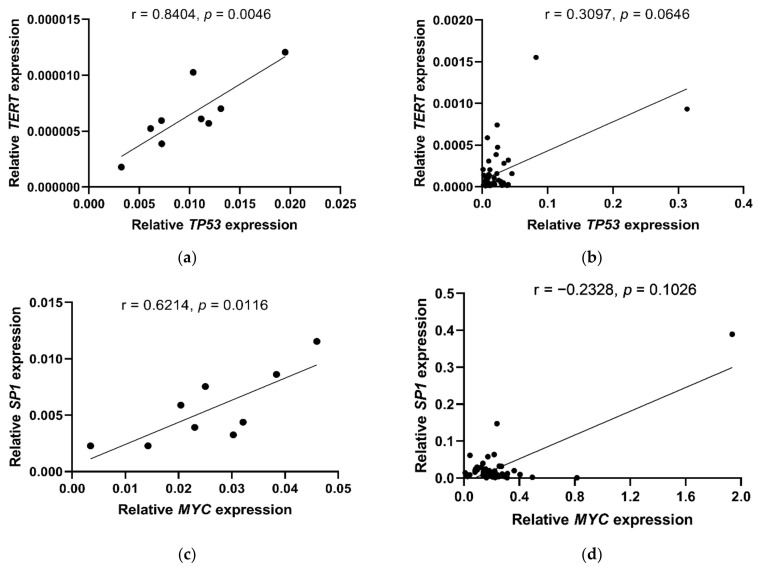
Relationships between expression of *TERT*, *TP53*, *SP1, MYC* genes observed in BC organoids (**a**,**c**) and BC patients (**b**,**d**). Statistical analysis was performed using the Pearson correlation (PC) test (**a**,**c**) and the Spearman r correlation test (**b**,**d**).

In this part of the study, we analyzed the relationships between *TERT*, *SP1*, *MYC* and *TP53* expression, *TERT* polymorphisms and telomere length in both patients with BC (*n* = 50) and BC organoids (*n* = 9). We observed a correlation between the relative expression of *TERT* and *TP53* in BC organoids (r = 0.8404, *p* = 0.0046; Figure 2a) and a trend towards this association in BC patients (r = 0.3097, *p* = 0.0646; Figure 2b). Moreover, we found a relationship between the expression of the *SP1* and *MYC* genes only in BC organoids (r = 0.6214, *p* = 0.0116; Figure 2c) and not in BC patients (r =−0.2328, *p* = 0.1026; Figure 2d).

**Figure 3:**
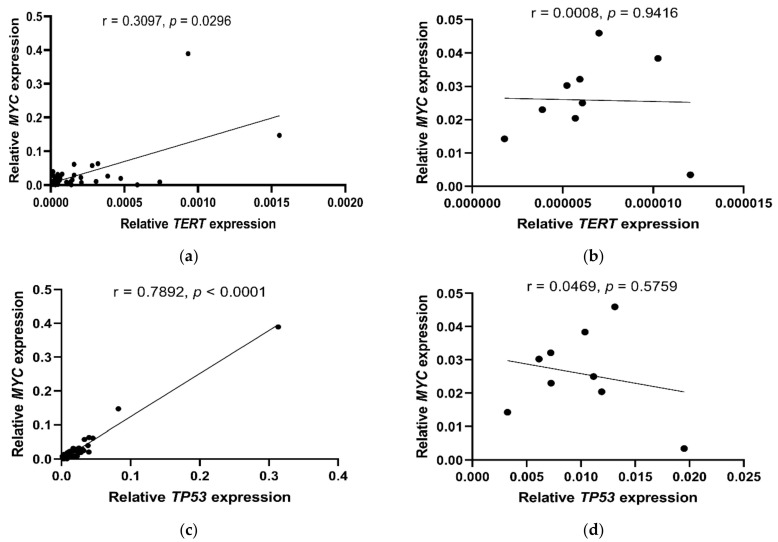
Relationships between the expression of *TERT*, *MYC* and *TP53* genes observed in the blood of BC patients (**a**,**c**) and BC organoids (**b**,**d**). Statistical analysis was performed using the Spearman r correlation test (**a**,**c**) and the Pearson correlation (PC) test (**b**,**d**).

A correlation between the expression of the *TERT* and *MYC* genes (r = 0.3097, *p* = 0.0296; Figure 3a) and between the expression of the *TP53* and *MYC* genes (r = 0.7892, *p* < 0.0001; Figure 3c) was also found, but only in BC patients and not in BC organoids (*TERT*/*MYC*: r = 0.0008, *p* = 0.9416; Figure 3b and *TP53*/*MYC*: r = 0.0469, *p* = 0.5759; Figure 3d).

Additionally, we only observed a trend toward associations between the relative gene expression of *TERT* (*p* = 0.0817) and *SP1* (*p* = 0.0774) in the context of BC subtypes (Luminal with *HER2* gene amplification, Luminal without *HER2* gene amplification and Triple Negative BC). We observed no such associations between *MYC* and *TP53* expressions. In addition, we observed a trend towards high estrogen receptor expression in patients with increased *TP53* expression (above average) (*p* = 0.0894). Moreover, BC patients with low *SP1* and *MYC* (below average) expression were characterized by high progesterone receptor expression (*p* = 0.0504 and *p* = 0.0897, respectively).

### 2.3. Genetic Variation in TERT, Telomere Length and Expression Level of TP53 and SP1 in BC Patients

**Figure 4:**
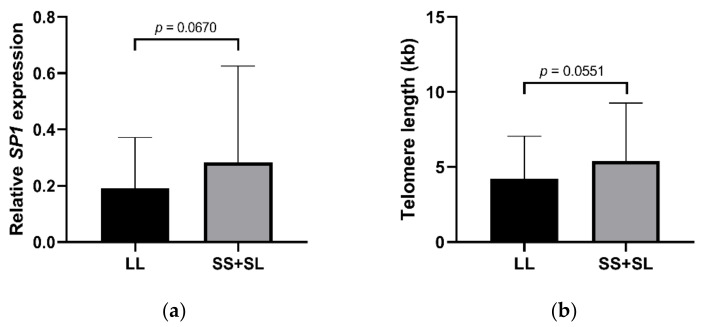
Associations between the *TERT* gene polymorphisms (10069690 and rs2735940), relative expression of the *TP53* gene (**a**,**b**), and telomere length (**c**,**d**) in patients with BC. The Mann–Whitney U test was employed to assess the significance of differences in the expression levels of *TP53* and rs10069690 (**a**) and in telomere length (**c**). The Kruskal–Wallis test with the Original FDR method of Benjamini and Hochberg was used to assess the significance of the relative expression of *TP53* and the genotypes in rs2735940 (**b**), as well as differences in telomere length (**d**).

We found a link between the expression level of *TP53* and *SP1*, the genetic variability in *TERT* and telomere length. BC patients with the *TERT* (rs10069690) *A* allele (*p* = 0.0266; Figure 4a) and patients with the *TERT* (rs2735940) *TT* genotype had the highest relative expression of the *TP53* gene (*p* = 0.0340; Figure 4b). Additionally, patients with the *TERT* (rs10069690) *A* allele had the longest telomeres (*p* = 0.0056) as compared to patients with the *GG* genotype (Figure 4c). However, patients with the *TT* genotype in *TERT* (rs2735940) did not have the longest telomeres compared to the other rs2735940 genotypes (*CC* vs. *CT*, *p* < 0.0001; *CC* vs. *TT*, *p* = 0.0562; *CT* vs. *TT*, *p* = 0.0074, Figure 4d). No significant associations were observed between either *TERT* rs2736100 (*GG* vs. *TG*, *p* = 0.5334; *GG* vs. *TT*, *p* = 0.3780; *TG* vs. *TT*, *p* = 0.7571) or *TERT* rs2853669 (*CC* vs. *CT*, *p* = 0.6034; *CC* vs. *TT*, *p* = 0.9039; *CT* vs. *TT*, *p* = 0.4233) and the relative expression levels of *TP53*. However, we observed that BC patients with the *GG* genotype rs2736100 had longer telomeres than women with the *TG* and *TT* genotypes (*GG* vs. *TG*, *p* < 0.0001; *GG* vs. *TT*, *p* = 0.0360; *TG* vs. *TT*, *p* = 0.0125).

**Figure 5:**
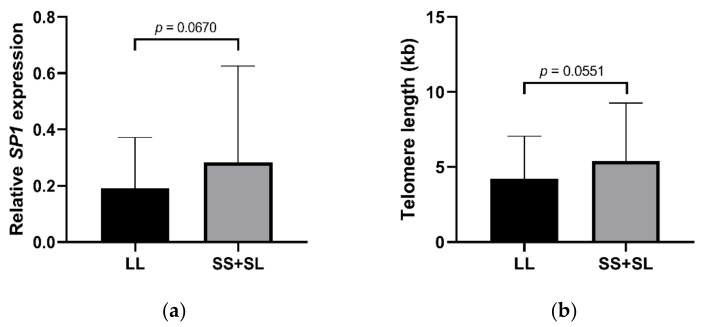
Relationship between the *TERT* VNTR-MNS16A polymorphism, relative *SP1* expression and telomere length. High relative expression of the *SP1* gene is associated with short allele (S) *TERT* VNTR-MNS16A (**a**), which was associated with long telomeres (**b**). The Mann-Whitney U test was employed to assess the significance of differences in the expression level of SP1 (**a**) and the differences of telomere length (**b**).

We noticed a trend for a relationship between *SP1* gene expression and the *TERT* VNTR-MNS16A gene polymorphism in BC patients. BC patients with SL (243/302, 243/333, 274/302) and SS (243/243, 274/274, 243/274) VNTR-MNS16A genotypes had a higher relative expression of *SP1* (*p* = 0.0670, Figure 5a) and the longest telomeres compared to the patients with LL genotypes (302/302, 302/333; *p* = 0.0551; Figure 5b).

### 2.4. Relationship between Gene Expression, TERT Genetic Variability, Telomere Length and Clinicopathological Hallmarks of Breast Cancer

In the present study, telomere length was measured in three independent groups: BC patients (*n* = 108), BC organoids (*n* = 9) and a group of healthy women (*n* = 100). We did not observe any significant differences between the telomere length in BC patients (4.95 ± 3.61 kb), healthy females (4.43 ± 2.26 kb) (Table 3) and BC organoids (3.75 ± 1.42 kb). We also did not notice any significant differences between the *TERT* genotypes (rs10069690, rs2735940, rs2736100, rs2853669, VNTR-MNS16A) and telomere length; the details are presented in Table S1 in the Supplementary Materials. In addition, no relationship was observed between telomere length and main clinical features (shown in Table 3).

It was observed that BC patients with an intermediate Ki67 proliferation index (25–50%) had the lowest relative expression of *TP53*—lower than patients with low (2–20%) and high (60–85%; *p* = 0.0221) levels of Ki67. Similarly, intermediate levels of Ki67 were characterized by the lowest expression of *TERT*, although this was not statistically significant. In addition, BC patients lacking the expression of the estrogen receptor tended to have lower relative *TP53* expression (*p* = 0.0894).

Analysis of the *TERT* polymorphisms showed that BC patients with *T* allele rs2736100 and *C* rs2735940 had more invasive tumors (assessed according to histologic grade (G), describing the aggressiveness and dynamics of tumor development) than patients with the *GG* genotype (rs2736100, *p* = 0.0008) and *TT* genotype (rs2735940, *p* = 0.0055). Moreover, *TERT* rs10069690 polymorphism showed that patients with the *A* allele had *HER2* gene amplification less frequently (*p* = 0.0268).

Additionally, BC patients with the *GG* genotype (rs2736100) had higher parathyroid hormone (PTH) levels (40.64 ± 16.78 pg/mL) than heterozygotes (28.11 ± 10.67 pg/mL; *p* = 0.0400) and *TT* homozygotes (35.36 ± 10.82 pg/mL; *p* = 0.0469). However, in the case of rs2735940 *TERT* polymorphism, it was observed that the heterozygous group of patients (28.73 ± 10.52 pg/mL) had the lowest concentration of PTH in the blood (*p* = 0.0408). For the *TERT* rs2853669 polymorphism, we only observed that BC patients with the *TT* genotype had higher blood estradiol levels (62.41 ± 61.90 pg/mL) compared to patients with *C* allele (25.80 ± 53.43 pg/mL; *p* = 0.0051).

The VNTR-MNS16A analysis showed that women with SS genotypes showed fewer invasive tumors classified by G feature than women with the LL or SL genotypes (*p* = 0.0181). Moreover, BC patients with heterozygous genotypes (SL) had less *HER2* amplification/overexpression than patients with homozygous genotypes (SS + LL) (*p* = 0.0097).

**Figure 6:**
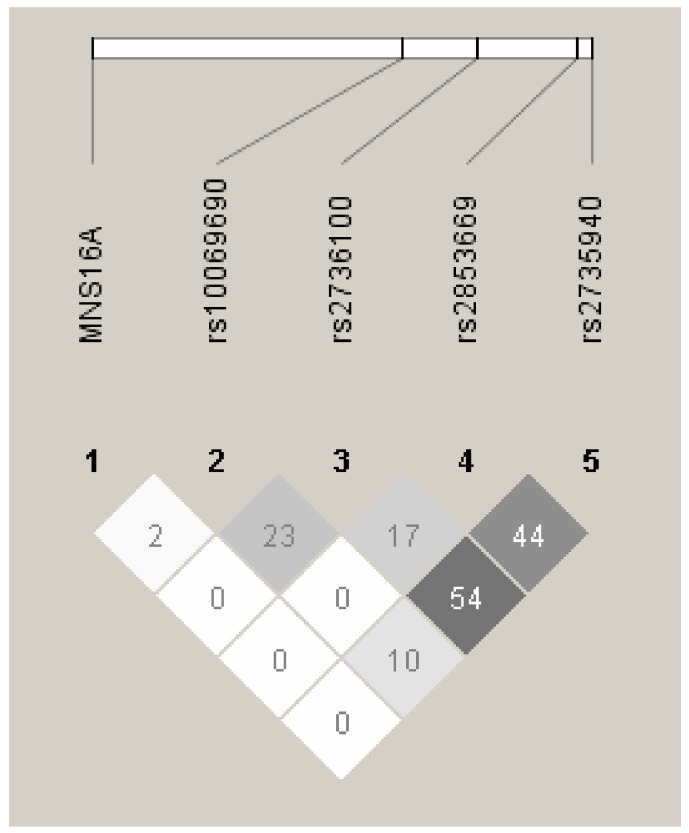
Analysis of linkage disequilibrium in patients with BC. Darker color shows higher r^2^ values, while the value shown in the squares is r2 × 102. LD was considered to be medium for r^2^ > 20 and strong for r^2^ > 80. The chart was created using the Haploview 4.2 software.

Additionally, we performed a linkage disequilibrium (LD) analysis and found that the two *TERT* SNPs (rs2736100 and rs2735940) were in a medium LD (r^2^ = 0.54 in BC patients; Figure 6). Moreover, three *TERT* SNPs (rs2736100, rs2853669 and rs2735940) were in a low LD (r^2^ = 0.10 in BC patients; Figure 6).

We observed an association between the two SNPs rs2853669 (allele *C*) and rs2735940 (genotype *TT*) by which BC patients with this combination of *C* allele and *TT* genotype presented higher levels of estradiol (54.58 ± 63.87 vs. 11.33 ± 11.04 pg/mL; *p* = 0.0484).

Additionally, further analysis showed that patients with the *TCC* (rs2736100, rs2853669 and rs2735940, respectively) were characterized by G feature (*p* = 0.0317). In addition, we observed a relationship between the combination of VNTR-MNS16A (L alleles) and *TCC* (rs2736100, rs2853669, rs2735940), showing that the BC patients with L*TCC* had more invasive tumors classified by G feature (*p* = 0.0029). Another combination showed that BC patients with the alleles *T* (rs2736100) and *A* (rs10069690) and with the SL genotype VNTR-MNS16A had a lower frequency of *HER2* amplification/overexpression (*p* = 0.0008).

## 3. Discussion

Breast cancer (BC) is characterized by a high level of gene heterogeneity. The determination of the molecular/biologic subtypes of BC is an important issue for the classification of this disease according to the status of hormone receptors (estrogen and progesterone), the human epidermal growth factor receptor 2 (HER2) and the Ki67 proliferation index. All these variables, together with the presence of somatic and/or germline mutations, are important for the prognosis and individual treatment of BC patients.

*TERT* appears to play a significant role in the description of BC [16,17]. Therefore, our research covered *TERT* gene expression and telomere length, as well as the expression of the transcription factors *MYC*, *SP1* and *TP53* detected at the mRNA level. Moreover, the genetic variability of the *TERT* gene was detected at the level of SNPs and VNTR in the context of telomere length and the clinical parameters of patients with BC.

The *TERT* gene is a major functional subunit of telomerase, and telomere length is critical to genome stability. Although the molecular mechanisms of *TERT* regulation have been described in detail in many cancers, it is not well understood in BC. It is known that many cellular processes are related to the presence of telomerase and are associated with apoptosis, uncontrolled cell division, the breakdown of the division cycle and the repair of damaged DNA. In this context, the choice to examine *TERT* and *TP53* gene expression seems justified. Molecular disruptions, e.g., mutation in both *TERT* and *TP53* genes, can alter expression and often lead to aberrant telomerase activation that can induce uncontrolled cell proliferation and oncogenesis in BC.

In the present study, we showed that BC patients with a high Ki67 proliferation index (60–80%) had an increased relative expression of the *TP53* gene compared to patients with a low Ki67 index (25–50%), who had a lower *TP53* expression. Similar data, although not statistically significant, were observed in the expression of the *TERT* gene, where high levels of Ki67 were characterized by high *TERT* expression (see the Results section).

The *TP53* gene is a well-known tumor suppressor gene—also known as the “guardian of the genome”—and its mutations may be considered a major biomarker of cancer. Its role has been associated with the regulation of apoptosis, cell cycle control and DNA damage repair processes [18].

We used cells from two sources, the blood of BC patients and BC organoids, to compare the expression of the *TERT, TP53, MYC* and *SP1* genes. We found correlations within the genes *TERT* and *TP53* in both of these two independent cell models.

It is important to know that under physiological conditions, the exposure of cells to various stress signals activates the p53 signaling pathway, allowing cells to activate several transcriptional programs, including cell cycle arrest, DNA repair, senescence and apoptosis, leading to the suppression of tumor growth [19,20]. It should be noted that all these processes are related to telomerase activity and the expression of *TERT*. Inactivation of the *TP53* gene caused by mutation drives cell invasion, proliferation and survival, thereby facilitating cancer progression and metastasis [21]. Marei et al. highlights recent advances in the understanding of the regulatory network by which mutant p53 proteins can modulate the molecular signaling pathways involved in cancer progression and/or protection [22]. A mutation in the *TP53* gene is detectable in approximately 50% of human breast, colon, lung, liver, prostate, bladder and skin cancers [23]. Many of these mutant p53 proteins are oncogenic and therefore modulate the ability of cancer cells to proliferate, escape apoptosis, invade and metastasize [24]. *TP53* has also been documented to be involved in the cellular responses to dysfunctional telomeres. Guièze et al. showed that patients with chronic lymphocytic leukemia (CLL) with impaired *TP53* have severe telomere dysfunction and high genomic instability. This group found that each type of *TP53* alteration was associated with very short telomeres and high *TERT* expression. Additionally, the disruption of *TP53* was characterized by the downregulation of the shelterin complex genes within the telomerase complex [25].

In our study, we observed a dual role of telomere length in the context of *TP53* expression and *TERT* variability. BC patients with the *TT* genotype in the *TERT* promoter (rs2735940) have a shorter telomere length and higher *TP53* expression. The opposite effect was observed in BC patients with *A* allele in intron 4 (rs10069690), who had a longer telomere length and higher *TP53* gene expression (see Figure 4).

The relationship between telomere length and BC risk is contradictory. First, no significant association was found between telomere length and the risk of BC [26,27,28]. Secondly, some recent reports have suggested that longer telomere lengths have been associated with an increased risk of BC [29,30]. Pellat et al.’s study strongly suggests that both telomere length and telomere related genes influence BC risk and that the tumor estrogen and progesterone receptors appear to be important modifiers of the associations with telomere related genes and BC risk [8]. However, other studies found that a shorter telomere length was associated with an increased risk of BC [31]. Shen et al. observed that, overall, telomere length was not significantly associated with the risk of BC. However, they noted that a shorter telomere length may be associated with an increased risk of BC in premenopausal women [31]. Additionally, Pooley et al. found a strong association between a shorter telomere length and BC risk [32]. One study found that both shorter and longer telomeres were associated with an increased risk of BC [33]. Oztas et al. reported that the rs2736100 *TERT C* allele is not associated with BC risk, but Aydin et al. observed the opposite [34,35]. De Souza Rodrigues et al. showed that the *TERT* variants rs2736098, rs10069690 and rs2853676 were associated with an increased risk of BC [17]. Additionally, it was observed that the VNTR-MNS16A influences the risk of BC in the Iranian population but not in the Greeks and Americans [36]. A meta-analysis by Aziz et al. did not show any significant associations of rs2853669 (located in the promoter region of *TERT*) genotypes in Caucasian BC patients [37]. Moreover, Varadi et al. found no clear association between a reduction in hereditary or occasional BC risk with rs2853669 in a cohort of Swedish patients [38].

In our study, we did not observe any significant differences in telomere length in BC patients with the *TERT* rs2736100 and rs2853669 alleles and genotypes. However, we noticed that patients with *TERT* VNTR-MNS16A with a short (S) allele had longer telomeres and higher expression of *SP1* mRNA (see Figure 5).

In an earlier study, Hofer et al. discussed the role of the VNTR-MNS16A polymorphism in the context of transcription factors and showed that transcription activity depends on various VNTR-MNS16A length variants presenting a different number of transcription factor binding sites for the GATA binding protein 1 [39].

In our study, we noticed a trend towards association between the expression of the *SP1* gene and the *TERT* VNTR-MNS16A gene polymorphism. Our BC patients with the S allele had a higher relative expression of *SP1* and longer telomeres than the patients with LL genotypes (see Figure 5a,b).

When we compared the genetic variability of *TERT* with the clinical data of the BC patients, we showed that BC patients with more invasive tumors were characterized by VNTR-MNS16A L allele and *TCC* (rs2736100, rs2853669 and rs2735940, respectively). Additionally, BC patients with the *T* allele (rs2736100), *A* allele (rs10069690) and SL genotype VNTR-MNS16A had a lower frequency of *HER2* amplification/overexpression. Moreover, patients with the *TT* genotype (rs2735940) and with the *C* allele (rs2853669) were characterized by lower levels of estradiol and higher levels of progesterone. Regarding the analysis of clinical data, Bojesen et al. showed that *TERT* rs10069690 is associated with a risk of estrogen receptor negative BC and BC in *BRCA1* mutation carriers, which is consistent with another observation that showed that most incidents of BC arising from *BRCA1* mutation carriers are estrogen receptor negative [40]. In our present study, we did not observe any significant association of genotype and risk of BC or *TERT* SNP with estrogen and progesterone receptor status and *BRCA1* mutation.

Interesting results documented by Gay-Bellile et al. presented the role of the *TERT T*349*C* (rs2853669) promoter polymorphism, which was not correlated with *TERT* expression, but carriers of the *TC* and *CC* genotypes had a significantly shorter disease-free survival [14]. Our present results confirm their observation of *TERT* expression in both BC patients and BC organoids, as *TERT* rs2853669 was not associated with *TERT* expression. Additionally, Gay-Bellile et al. showed that *TERT* gains found in 15–25% of cases were strongly correlated with increased *TERT* mRNA expression and worse patient prognosis in terms of disease-free and overall survival [14].

Our study provides definitive evidence of the genetic control of telomere length by some of the genetic variants in the *TERT* locus (e.g., VNTR-MNS16A, rs2735940, rs10069690). Additionally, we showed that *TERT* genetic variants could be potential prognostic biomarkers of BC associated with tumor invasiveness. Given the limitations of this study, future studies with a larger sample size to validate the current findings are needed, as well as functional studies to reveal the role of the *TERT* gene polymorphism and mRNA expression in BC carcinogenesis.

## 4. Materials and Methods

### 4.1. Patients and Controls

The study included 108 Polish women (age range at diagnosis: 32–86 years, median 61 years) treated for invasive breast cancer at the Lower Silesian Oncology, Pulmonology and Hematology Center (Wroclaw, Poland). The blood samples were collected at diagnosis after obtaining informed consent from the patients. All methods were according to the Declaration of Helsinki. The approval of the Bioethical Committee of Wroclaw Medical University was obtained for the study (No. KB—808/2019). Additionally, 100 healthy blood donors (age range: 18–59, median 21 years) served as a control group for the study of *TERT* polymorphisms and telomere length. Relationships between telomere length and the various clinical parameters of the studied group are presented in Table 3. Our study group included 8 women with different variants of germline mutations in the *BRCA1* (c.181T > G (p.Cys61Gly); c.5266dupC)), *BRCA2* (c.9227G > A; c.10202C > T (p.Thr3401Met)), *CHEK2* (c.444 + 1G > A (IVS3 + 1G > A)) and *PALB2* (c.172_175del) genes, 74 BC patients without these germline mutations and 26 BC patients who were not tested for germline mutations. All BC patients and control subjects were Polish Caucasians recruited from the population of Lower Silesia (south-western province of Poland, ≈ 2.9 M population in 2019).

### 4.2. Breast Cancer Organoids

The sample was the tissue from eight BC patients (age range at diagnosis: 37–76 years old, median 47 years) with infiltrating duct carcinoma [(NOS) 8500/3] G1, 2, 3 before radiotherapy, chemotherapy and other treatment. The tissues were delivered as a postoperational material from the Gdynia Oncology Center of the Polish Red Cross Maritime Hospital. The human material was sampled according to the local bioethical commission guidelines (but no particular permission was required since the material was obtained within regular surgery operations removing carcinoma). However, according to the bioethical commission guidelines, the informed consent of the patient was necessary and was obtained each time. The tissues were then washed using phosphate buffer saline (PBS 1 ×, Gibco, Waltham, MA, USA) and preserved in the transfer medium consisting of DMEM/F12, +10% Fetal Bovine Serum (FBS, Sigma-Aldrich, Saint Louis, MO, USA) + 100 µg/mL Penicillin/Streptomycin + 5 µg/mL Piramycin + 50 U/mL Polymyxin B before being isolated. After that, the tissues were washed with 1 × PBS in a Petri dish and then cut into small pieces using a surgical scalpel. The sample fragments were washed again with 1 × PBS, inserted into a 15 mL falcon tube (Sigma-Aldrich, Saint Louis, MO, USA) containing the mixed enzyme solution and then incubated for 16 h, 300 rpm, 37 °C. After incubation, the samples were filtered using 100 μm and 40 μm cell strainer (Corning, New York, NY, USA) and then centrifuged at 600× *g* for 5 min. The supernatant was discarded, and the pellet containing tissue fragments was washed with 1 × PBS and centrifuged at 600× *g* for 5 min. One part of the material was frozen using RNA later (Thermo Fisher Scientific, Waltham, MA, USA) or 50% DMEM/F12 + 44% FBS + 6% Dimethyl sulfoxide (DMSO, Sigma-Aldrich, Saint Louis, MO, USA) and Nunc type freezing ampoules (Thermo Fisher Scientific, Waltham, MA, USA). The remnant pellet was then resuspended with the culture initiation media and cultured in a 6-well plate (37 °C, 5% CO_2_) for 48 h. Afterwards, the media mix was removed and the stimulation medium was added, which was replaced every 3 days. Next, the cells were transferred into T75 flasks and cultured in the stimulation medium until reaching a confluence of 80%. The cultured cells were then detached using trypsin (Sigma-Aldrich, Saint Louis, MO, USA) and incubated for 1–3 min at 37 °C, and medium containing FBS was added to neutralize trypsin. The detached cells were centrifuged at 600× *g* for 5 min at room temperature. The cells were counted using a Z series Coulter Counter by Beckman Coulter, Indianapolis, IN, USA. Eventually, the cells were frozen using RNA later or 75% stimulation medium + 15% FBS + 10% DMSO and Nunc type freezing ampoules. The ampoules were stored at −80 °C until further analysis.

### 4.3. DNA Extraction

Genomic DNA was isolated from 200 μL of peripheral blood taken on EDTA using the NucleoSpin Blood kit (MACHEREY-NAGEL GmbH & Co. KG, Dueren, Germany) according to the manufacturer’s instructions. Genomic DNA from the BC organoids was isolated using NucleoSpin Tissue XS kits (MACHEREY-NAGEL GmbH & Co. KG, Dueren, Germany). DNA concentration and purity were quantified on a DeNovix DS-11 spectrophotometer (DeNovix Inc., Wilmington, DE, USA). The isolated DNA was then stored at −20 °C until *TERT* genotyping and evaluation of the telomere length in patients with BC and BC organoids.

### 4.4. Genotyping of TERT Gene Polymorphisms

The selection of the studied single nucleotide polymorphisms (SNPs) within the *TERT* gene was based on results of the SNP Function Prediction tool available on the website of the National Institute of Environmental Health Sciences (NCBI Database), as well as other auxiliary databases (https://snpinfo.niehs.nih.gov/snpinfo/snpfunc.html (accessed on 2 February 2022); https://www.ncbi.nlm.nih.gov/snp/ (accessed on 2 February 2022); https://www.ensembl.org/index.html (accessed on 2 February 2022). The following criteria were used: minor allele frequency in Caucasians above 10%, change in RNA and/or amino acid chain, potential splicing site and/or miRNA binding site.

Based on the above criteria, four *TERT* SNPs were selected for the study: rs10069690 (G > A) located in intron 4; rs2736100 (G > T) located in intron 2; rs2853669 (T > C) and rs2735940 (T > C), both located in the promoter region at −245 bp (Ets2 binding site) and 1327 bp upstream of the transcription start site, respectively. The *TERT* polymorphisms were determined by LightSNiP typing assays (TIB MOLBIOL, Berlin, Germany) using quantitative polymerase chain reaction (qPCR). Amplifications were performed on a LightCycler480 II Real-Time PCR system (Roche Diagnostics International AG, Rotkreuz, Switzerland) according to the recommendations of the manufacturer. The PCR conditions were as follows: 95 °C for 10 min followed by 45 cycles of 95 °C for 10 s, 60 °C for 10 s and 72 °C for 15 s. PCR was followed by one cycle of 95 °C for 30 s, 40 °C for 2 min and gradual melting from 75 °C to 40 °C.

### 4.5. VNTR-MNS16A Genotyping of the TERT Gene

The presence of the VNTR-MNS16A *TERT* gene polymorphism was assessed in BC patients and in healthy women by PCR amplification followed by electrophoresis in sequencing gel, as described by Wysoczanska et al. [41]. PCR was performed in a 2720 Thermal Cycler instrument (Applied Biosystems, Foster City, CA, USA) using the forward and reverse primer sequences (5′-AGGATTCTGATCTCTGAAGGGTG-3′ and 5′-TAMRA-TCTGCCTGAGGAAGGACGTATG-3′) prepared by Genomed (Warsaw, Poland). The amplification procedure included an initial denaturation step for 5 min at 95 °C, followed by 35 cycles: 30 s at 95 °C, 30 s at 65 °C, 30 s at 72 °C and a final extension step for 10 min at 72 °C. The PCR products were diluted with formamide and a GeneScan™500 ROX™ dye Size Standard (Applied Biosystems, Foster City, CA, USA). The samples were denatured at 95 °C for 5 min and analyzed on the 3500 Genetic Analyzer (Applied Biosystems, Foster City, CA, USA) with an eight-capillary system filled with POP7 polymer (Applied Biosystems, Foster City, CA, USA). The alleles were identified using the GeneMapper software version 4.2 (Applied Biosystems, Foster City, CA, USA).

### 4.6. Quantification of Telomere Length

Mean telomere length was measured in the genomic DNA samples of 108 BC patients, 100 controls and 9 BC organoids. The DNA samples were diluted with nuclease-free water to a concentration of 5 ng/mL. Telomere length measurements were performed on a LightCycler480 II Real-Time PCR system (Roche Diagnostics International, Rotkreuz, Switzerland) using qPCR test kits (ScienCell’s Absolute Human Telomere Length Quantification qPCR Assay Kit [AHTLQ], Carlsbad, CA, USA), as previously described by Dratwa et al. [42]. The PCR conditions were as follows: 95 °C for 10 min followed by 32 cycles of 95 °C for 20 s, 52 °C for 20 s and 72 °C for 45 s. Data analysis was conducted according to the manufacturer’s instructions. All reactions were run in three replicates.

### 4.7. Extraction of RNA, Reverse Transcription and TERT, SP1, MYC and TP53 Genes Expression Study

The RNA of 50 patients with BC and 9 BC organoids was extracted from 10^6^ cells suspended in RNA Extracol (EURx, Gdansk, Poland) or RNA later (Thermo Fisher Scientific, Waltham, MA, USA) according to the manufacturer’s instructions. RNA purity and integrity were verified on a DeNovix DS-11 spectrophotometer (DeNovix Inc., Wilmington, DE, USA) and gel electrophoresis. A total of 1 μg/μL of the isolated RNA was used for the reverse transcription reaction. cDNA was synthesized using the High Capacity cDNA Reverse Transcriptase kit (Applied Biosystems™, Foster City, CA, USA), and 0.5 μL of RNase Inhibitor (Applied Biosystems™, Foster City, CA, USA) was added per sample to convert the extracted and purified RNA into cDNA. The conversion step was performed on a SimpliAmp™ Thermal Cycler (Applied Biosystems^®^, Foster City, CA, USA). After this step, the samples were stored in a freezer at −20 °C until further use.

Four genes were included in the expression experiments: *TERT* (Hs_00972,650_m1), *SP1* (Hs_00916521_m1), *MYC* (Hs_00153408_m1) and *TP53* (Hs_01034249_m1). *GAPDH* (Hs02786624_g1) and *ACTB* (Hs_01060665_g1) were used as housekeeping genes to normalize RNA expression data. TaqMan^®^ Gene expression assays were used for detection (Applied Biosystems Foster City, CA, USA), and qPCR was performed using the LightCycler 480 II Real-Time PCR system (Roche Diagnostics International, Rotkreuz, Switzerland). The following protocol was used for each PCR sample: 5 μL of cDNA, 1 μL (20×) each primer/probe, 10 μL (2×) of TaqMan^®^ Gene Expression Master Mix (Applied Biosystems™, Foster City, CA, USA), 4 μL of ultra-pure water. Amplification was performed under the following conditions: initial denaturation for 10 min at 95 °C was followed by 40 cycles of denaturation for 15 s at 95 °C and annealing for 1 min at 60 °C. Relative genes’ expression levels were calculated by the 2^−ΔCT^ method. Each sample was analyzed in triplicate to validate the technique and CT values, according to the international standards for the evaluation of gene expression by real-time PCR.

### 4.8. Statistical Analysis

The null hypothesis that there is no difference between the frequency of alleles and genotypes between patients and controls was verified with the Fisher’s exact test, calculated using the online tool http://vassarstats.net/tab2x2.htm (version as of 2 February 2022). In each experiment, the normality of the data was verified with the Shapiro-Wilk test. The remaining statistical analyses of the differences between the groups were performed using one-way analysis of variance (ANOVA) to determine the significance of intergroup differences, and the obtained *p*-values were corrected by the Benjamini and Hochberg method. Taking into account that the distribution of some data deviates from the normal distribution, the non-parametric U Mann–Whitney test was performed for the comparison of telomere lengths and gene expression. The correlations were statistically evaluated using the Pearson correlation (PC) test or the Spearman r test. The statistical calculations were performed by the GraphPad Prism software (GraphPad Software, La Jolla, CA, USA, version 8.0.1) and the Real Statistics Resource Pack for Microsoft Excel 2019 (version 16.0.10369.20032, Microsoft Corporation, Redmont, Washington, DC, USA). The probability (*p*) values < 0.05 were considered statistically significant, while the trend index was between 0.05 and 0.10.

## Figures and Tables

**Table 1:** Distribution of *TERT* genotypes in our group of patients with BC, the control group and the European population.

*TERT* Genetic Polymorphism	Genotype	BC Patients Frequency	Control Group Frequency	EUR Population Frequency
rs10069690 (intron 4)	*GG* *AG* *AA*	59 (53.2%) 48 (43.2%) 4 (3.6%)	46 (48.4%) 42 (44.2%) 7 (7.4%)	265 (52.7%) 198 (39.4%) 40 (8.0%)
rs2735940 (promoter region)	*CC* *TC* *TT*	35 (30.9%) 54 (47.8%) 24 (21.2%)	22 (23.2%) 54 (56.8%) 19 (20.0%)	127 (25.2%) 238 (47.3%) 138 (27.4%)
rs2736100 (intron 2)	*GG* *TG* *TT*	28 (23.7%) 52 (44.1%) 38 (32.2%)	24 (22.6%) 52 (49.1%) 30 (28,3%)	134 (26.6%) 234 (46.5%) 135 (26.8%)
rs2853669 (promoter region)	*CC* *CT* TT	11 (9.8%) 40 (35.7%) 61 (54.5%)	8 (7.5%) 39 (36.8%) 59 (55.7%)	49 (9.7%) 192 (38.2%) 262 (52.1%)

**Table 2:** *TERT* VNTR-MNS16A genotype distribution and telomere length in BC patients and healthy controls.

*TERT* VNTR-MNS16A Genotypes	BC Patients (*n*)	Telomere Length (Mean ± Std. Deviation) [kb]	Health Controls (*n*)	Telomere Length (Mean ± Std. Deviation) [kb]
Long VNTR-MNS16A (LL)				
302/302	41	4.21 ± 2.85	36	3.79 ± 1.59
302/333	2		3	
Short/Long VNTR-MNS16A (SL)				
243/302	40	4.95 ± 3.05	46	4.66 ± 1.48
243/333	1		2	
274/302	5		6	
Short VNTR-MNS16A (SS)				
243/243	11	6.72 ± 5.48	6	7.80 ± 5.33
274/274	3		not detected	
243/274	2		1	

**Table 3:** Relationships between telomere length and various clinical parameters in patients with BC.

	BC Patients	*n*	Telomere Length Median (IQR) [kb]	*p*-Value
Age (range)	18–59 years	108	5.53 (2.68–5.94)	0.4903
Estrogen receptor	Positive Negative	93 7	3.44 (2.64–5.76) 5.02 (3.15–5.87)	0.2502
HER2 amplification	Positive Negative	15 83	4.24 (2.78–6.99) 3.36 (2.64–5.57)	0.3299
Progesterone receptor	Positive Negative	88 14	3.53 (2.65–5.92) 5.08 (2.93–6.46)	0.3261
Molecular subtypes	Luminal with HER2 gene amplification	15	4.70 (2.75–7.09)	0.4797
	Luminal without HER2 gene amplification	76	3.37 (2.62–5.86)	
	Triple Negative BC	7	5.02 (2.93–5.86)	
UICC TNM stage	I II III	48 40 5	3.83 (2.41–5.67) 3.27 (2.69–6.28) 3.26 (3.13–4.08)	0.9433
Pathologic lymph nodes status	pN0 pN+	77 24	3.39 (2.64–5.15) 4.12 (2.71–5.94)	0.4666
Germline mutation (*BRCA1*, *BRCA2*, *CHEK2*, *PALB2*)	Positive Negative	8 74	6.66 (2.74–6.05) 4.02 (2.66–6.28)	0.6727

## Data Availability

The data presented in this study are available upon request from the corresponding author. The data are not publicly available due to privacy or ethical restrictions.

## Supplementary Materials

The following supporting information can be downloaded at: https://www.mdpi.com/article/10.3390/ijms23095164/s1.
