# Supplementary material for: Relationship between Telomere Length, TERT Genetic Variability and TERT, TP53, SP1, MYC Gene Co-Expression in the Clinicopathological Profile of Breast Cancer
Source: Int J Mol Sci. 2022 May 5;23(9):5164. doi: 10.3390/ijms23095164 (PMC9102200; doi:10.3390/ijms23095164)
Supplement: Supplementary file 1 [file ijms-23-05164-s001.zip › Table S1.pdf]

Table S1. Distribution of *TERT* genotypes, telomere length and *TERT* expression in our group of patients with BC.

Genotypes	Genotypes distribution (n)	Telomere length median [IQR] [kb]	Relative *TERT* gene expression (n)	Relative *TERT* gene expression median [IQR] ($2^{-\Delta CT}$)	Genetic models (*p*-value)		
					Dominant	Recessive	Co-dominant
**rs10069690 (intron 4)**							
GG	59	3.60 [2.69-6.21]	26	$5.06\times10^{-5}[3.36\times10^{-5}-1.36\times10^{-4}]$	*AA+AG* vs *GG*	*AA* vs *AG+GG*	*AA* vs *AG* vs *GG*
AG	48	3.46 [2.70-6.03]	22	$5.63\times10^{-5}[2.11\times10^{-5}-2.87\times10^{-4}]$	(*p* = 0.759)	(*p* = 0.423)	(*p* = 0.702)
AA	4	3.64 [2.13-4.71]	-	-			
**rs2735940 (promoter region)**							
CC	35	3.14 [2.68-6.11]	15	$4.06\times10^{-5}[1.79\times10^{-5}-1.36\times10^{-4}]$	*TT+TC* vs *CC*	*TT* vs *CC+CT*	*TT* vs *TC* vs *CC*
TC	54	3.83 [2.69-6.11]	21	$5.88\times10^{-5}[2.75\times10^{-5}-2.33\times10^{-4}]$	(*p* = 0.930)	(*p* = 0.443)	(*p* = 0.684)
TT	24	3.06 [2.50-5.30]	13	$4.88\times10^{-5}[2.72\times10^{-5}-2.44\times10^{-4}]$			
**rs2736100 (intron 2)**							
GG	28	3.53 [2.63-5.14]	15	$4.18\times10^{-5}[2.21\times10^{-5}-2.08\times10^{-4}]$	*TT* vs *GG+TG*	*TT +TG* vs *GG*	*TT* vs *TG* vs *GG*
TG	52	3.39 [2.64-6.52]	19	$5.69\times10^{-5}[2.74\times10^{-5}-1.58\times10^{-4}]$	(*p* = 0.485)	(*p* = 0.715)	(*p* = 0.776)
TT	38	3.78 [2.81-5.84]	15	$4.88\times10^{-5}[3.57\times10^{-5}-1.39\times10^{-4}]$			
**rs2853669 (promoter region)**							
CC	11	3.36 [2.35-5.07]	9	$2.81\times10^{-5}[1.94\times10^{-5}-1.83\times10^{-4}]$	*TT* vs *TC +CC*	*CC* vs *TC+TT*	*CC* vs *TC* vs *TT*
CT	40	3.51 [2.67-6.06]	13	$1.06\times10^{-5}[4.67\times10^{-5}-3.14\times10^{-4}]$	(*p* = 0.622)	(*p* = 0.569)	(*p* = 0.807)
TT	61	3.56 [2.74-6.11]	26	$4.76*10^{-5}[1.76\times10^{-5}-1.40\times10^{-4}]$			
